# Criterion and Divergent Validity of the Sexual Minority Adolescent Stress Inventory

**DOI:** 10.3389/fpsyg.2017.02057

**Published:** 2017-11-28

**Authors:** Jeremy T. Goldbach, Sheree M. Schrager, Mary R. Mamey

**Affiliations:** ^1^Suzanne Dworak-Peck School of Social Work, University of Southern California, Los Angeles, CA, United States; ^2^Division of Hospital Medicine, Children's Hospital Los Angeles, Los Angeles, CA, United States; ^3^Office of Research and Sponsored Programs, California State University, Northridge, CA, United States

**Keywords:** adolescents, LGBT, minority stress, mental health, behavioral health

## Abstract

Sexual minority adolescents (SMA) consistently report health disparities compared to their heterosexual counterparts, yet the underlying mechanisms of these negative health outcomes remain unclear. The predominant explanatory model is the minority stress theory; however, this model was developed largely with adults, and no valid and comprehensive measure of minority stress has been developed for adolescents. The present study validated a newly developed instrument to measure minority stress among racially and ethnically diverse SMA. A sample of 346 SMA aged 14–17 was recruited and surveyed between February 2015 and July 2016. The focal measure of interest was the 64-item, 11-factor Sexual Minority Adolescent Stress Inventory (SMASI) developed in the initial phase of this study. Criterion validation measures included measures of depressive symptoms, suicidality and self-harm, youth problem behaviors, and substance use; the general Adolescent Stress Questionnaire (ASQ) was included as a measure of divergent validity. Analyses included Pearson and tetrachoric correlations to establish criterion and divergent validity and structural equation modeling to assess the explanatory utility of the SMASI relative to the ASQ. SMASI scores were significantly associated with all outcomes but only moderately associated with the ASQ (*r* = −0.13 to 0.51). Analyses revealed significant associations of a latent minority stress variable with both proximal and distal health outcomes beyond the variation explained by general stress. Results show that the SMASI is the first instrument to validly measure minority stress among SMA.

## Introduction

Recent analyses of the National Longitudinal Study of Adolescent to Adult Health (Add Health) suggested that upward of 10% of youth do not identify as exclusively heterosexual and all sexual minority adolescent (SMA) subgroups (gay, lesbian, bisexual, mostly heterosexual) report health disparities (Marshal et al., 2013). SMA are 3 to 4 times more likely to meet criteria for an internalizing disorder and 2 to 5 times more likely to meet criteria for externalizing disorders than their heterosexual peers (Fergusson et al., 1999). This includes higher rates of internalizing psychopathology such as depression, anxiety, and self-harm (Anhalt and Morris, 1998; Haas et al., 2011; Hendricks and Testa, 2012) and substance use (Marshal et al., 2009). SMA are more than twice as likely to have attempted suicide compared to their heterosexual peers (Russell and Joyner, 2001; Schulenberg et al., 2001; Hatzenbuehler, 2011). A recent meta-analysis found SMA are almost 3 times more likely to report a history of suicidality and 5 times more likely to make an attempt than their peers (Marshal et al., 2011). These youth also more frequently report lower academic achievement (D'Augelli et al., 2002; Kosciw et al., 2012; Poteat et al., 2014) and higher rates of eating disorders and obesity (Austin et al., 2013) than their heterosexual peers. When these disparities occur in adolescence, they can negatively influence a lifelong trajectory of health (Baer, 1993).

### Minority stress

The primary framework for understanding mental health disparities among SMA is minority stress theory (Rosario et al., 2002; Meyer, 2003; Hatzenbuehler et al., 2009), which has been endorsed by the Centers for Disease Control and Prevention (2011), the Institute of Medicine (2011; now the National Academy of Medicine), and Healthy People 2020 (2011). Minority stress theory (Meyer, 2003) posits that an array of unique and chronic psychosocial stressors affects sexual minorities and contributes to negative behavioral health patterns. These include both distal stressors in the environment (e.g., prejudicial events, discrimination, and violence) and proximal stressors internal to the individual (e.g., expectations of rejection, concealment, and internalized homophobia).

Given that stigmatizing experiences can disrupt the achievement of developmental tasks during adolescence and contribute to negative outcomes (Radkowsky and Siegel, 1997), scholars have become increasingly interested in its impact during adolescence (Goldbach and Gibbs, 2015). Dozens of cross-sectional studies have attributed minority stress to behavioral health outcomes among SMA, including disclosure of sexual identity to family and peers (Remafedi et al., 1998; Almeida et al., 2009; D'Augelli et al., 2010; Haas et al., 2011), fear of becoming homeless upon disclosure (Rice et al., 2013), in-school victimization (i.e., bullying) by both students and faculty members (Russell et al., 2011; Toomey et al., 2011), and experiences of violence (D'Augelli et al., 2010; Friedman et al., 2011; Kosciw et al., 2013). To our knowledge, only six studies (with four unique samples) have examined the relationship between minority stress and later behavioral health outcomes in adolescents using a longitudinal design. These studies have found mixed support that minority stress was associated with future reporting of emotional distress, depression, suicide attempt (Rosario et al., 2002; Burton et al., 2013; Mustanski and Liu, 2013; Birkett et al., 2015), cigarette smoking, and substance use (Newcomb et al., 2014; Dermody et al., 2016). However, this work relied largely on brief measures of minority stress—from just four to 11 items—and the creation of *ad hoc* measures is standard practice (Morrison et al., 2016).

### Psychometric assessment of minority stress during adolescence

Despite wide acknowledgement that minority stress is the likely driver of mental health disparities, rigorous measures of minority stress remain nonexistent. Recently, Morrison et al. (2016) completed a psychometric review of measures assessing discrimination against sexual minorities. Even without specific restriction to adolescence, their findings add further confirmation of the lack of psychometrically sound instruments for accurately observing minority stress. In their review of 162 articles, nearly all included measures had suboptimal psychometric properties. Further, most were not created in collaboration with sexual minorities; possessed a very small number of items, thus insufficiently representing the domain of interest; and adapted from scales originally intended to measure other types of discrimination (e.g., gender- and race-based). As they accurately concluded, “in the absence of this type of (gold standard) rigorous assessment, it will be impossible to formulate a coherent picture of sexual minorities' discriminatory experiences and the relationships between discrimination and indices of … psychological wellness” (Morrison et al., 2016, p. 1096).

For adolescents, this measurement concern may be further compounded by developmental life stage. In general, the disproportionate attention to adult samples in sexual minority research (particularly young men who have sex with men) has been described elsewhere (e.g., Institute of Medicine, 2011). However, from a developmental perspective, adolescence is a critical period during which individuals establish long-term trajectories of health and are often still solidifying their sexual identities (Mustanski et al., 2013). Further, the stress process for all adolescents (regardless of sexual orientation) is associated with multiple factors related to peer relationships and pressure, demands from school and other responsibilities, lack of family bonding, family conflicts, and physical and psychological changes along with family and adolescent expectations for the future (Robson and Cook, 1995; De La Rosa, 2002; Guinn and Vincent, 2002; Goldbach and Gibbs, 2015). Thus understanding how minority-related stress experiences may discriminate from general adolescent stress is principal. This problem was illustrated in a recent meta-analysis of minority stress and substance use among SMA (Goldbach et al., 2014), in which the authors found that few studies used empirically validated measures; most measures had been developed through small investigator-led samples lacking racial and ethnic diversity or were developed with adults; and no studies had clearly operationalized minority stress domains for SMA. Further, studies that used measures of “general distress” found large correlations to substance use (*r* = 0.60), whereas those that used measures of “gay-related stress” found a much weaker relationship (*r* = 0.24). Thus currently available measures of SMA stress may not accurately capture the stress–outcome relationship; although general measures may capture more breadth, they do not allow us to understand differences between common developmental stressors and those specifically associated with sexual orientation.

In short, valid and reliable measurement is a necessary antecedent in the development of explanatory research and intervention efforts (Sandler et al., 1997) and accurate clinical assessment and appraisal of mental health needs (Watkins et al., 1995; Groth-Marnat, 2009). To address this gap in the study of minority stress among adolescents, the present study sought to determine the criterion and divergent validity of a newly developed measure of minority stress for adolescents, the Sexual Minority Adolescent Stress Inventory [SMASI; (blinded for review)]. The intent is to provide the first comprehensive measure of minority stress to the field for use in future research and practice settings.

## Materials and methods

### Participants and procedure

The sample consisted of 346 adolescents aged 14–17 who self-identified as either male or female and as being gay, lesbian, or bisexual. The full recruitment and data collection strategy for the study is described elsewhere (blinded for review); in brief, an initial set of individuals were recruited to participate in the study through in-person interviews and targeted online social media (e.g., Facebook, Reddit discussion forums). Subsequently, in an effort to improve generalizability, youth were provided a referral code that they could share with their peers using respondent driven sampling (RDS; Heckathorn, 1997). 63.6% of youth in our sample were recruited through the RDS process. A screener questionnaire was given to determine whether the person met the inclusion criteria before moving forward with the survey. Eligible participants answered questions for the SMASI, Adolescent Stress Questionnaire (ASQ; Byrne et al., 2007), revised Youth Self Report (R-YSR; Ivanova et al., 2007), 4-item Center for Epidemiologic Studies Depression Scale (CESD-4; Melchior et al., 1993), suicidality (Goldbach et al., 2017), and substance use (Centers for Disease Control and Prevention, 2010). The study received approval from an affiliated institutional review board.

### Measures

#### Sexual minority adolescent stress inventory

The SMASI was developed through an iterative process, described in full elsewhere (blinded for review), in which items were initially derived from qualitative interviews with SMA and revised and expanded during a modified Delphi process. A candidate item set of 102 items was included in the present study. Exploratory factor analysis on the lifetime endorsement of these items yielded an initial set of 12 stable factors comprising 72 items. Item response theory (IRT) techniques, including estimation of discrimination, and difficulty parameters and item characteristic curves, were applied overall and across demographic subgroups, to arrive at a final measure demonstrating configural and scalar invariance for gender, age, sexual identity, and race/ethnicity.

The main SMASI instrument produced by this analytic approach is a 10-factor measure composed of 54 items [blinded for review]. The inclusion of an optional 10-item factor describing minority stress experiences related to employment (termed *work*, e.g., “My workplace does not protect LGBTQ employees”) resulted in a comprehensive 11-factor measure with 64 items. Subscales of the main measure include items representing *social marginalization* (“Other youth refuse to hang out with me because I am LGBTQ,” 8 items); *family rejection* experiences (“I have to lie to my family about being LGBTQ,” 11 items); beliefs associated with *internalized homonegativity* (“There are times when I do not want to be LGBTQ,” 7 items); difficulties with *identity management* (“I am having trouble accepting that I am LGBTQ,” 3 items); experiences of a *homonegative climate* (“It's hard to be an LGBTQ person at my school,” 4 items); *intersectionality* between multiple minority statuses (“Other people who are in my racial/ethnic community judge me for being LGBTQ,” 3 items); *negative disclosure experiences* (“I was forced to come out to someone because I got ‘caught’, ” 5 items); stress associated with *religion* (“My family is part of a religion that has homophobic beliefs,” 5 items); *negative expectancies* about future treatment (“I expect people to reject me when they find out that I am LGBTQ,” 3 items); and experiences of *homonegative communication* (“My friends make jokes about LGBTQ people,” 5 items).

Lifetime and 30-day factor scores were created by calculating the percentages of endorsed items in each of these 11 factors at any time point, or specifically within the last 30 days, respectively, allowing for comparison of effects across subscales with differing numbers of items. For example, a person who endorsed four of five items in a particular subscale had a score of 80% for that subscale. Total scores were also calculated for both lifetime and 30-day stressors by summing the total number of items endorsed within each time frame for all factors except work (theoretical range: 0–54), because work items were only asked of participants who indicated an employment history in the demographic questions. As reported elsewhere (blinded for review), the total scale and subscales demonstrated good to excellent reliability (scale α = 0.98; subscale α = 0.75–0.96). The complete SMASI measure is presented in Supplementary Table 1.

#### General adolescent stress

The ASQ is a 10-factor measure consisting of 56 items that measure general stress in adolescents (Byrne et al., 2007). Participants are first asked to indicate whether they experienced a given situation during the past year (0 = no; 1 = yes). Participants who experienced the situation are then asked to rate the stressfulness of the situation on a 5-point scale (1 = not at all stressful to 5 = very stressful). Subscale scores were created by multiplying the binary items with the stress scale prompt. Per the instructions of the ASQ, those who had never experienced each situation received the same rating as those who had experienced the situation but reported it was not at all stressful. This resulted in a 5-point Likert-type scale ranging from 1 (not at all stressful is irrelevant to me) to 5 (very stressful). Subscales for the ASQ were created by summing item scores in each factor. These 10 subscales were used in the divergent validation analyses; the ASQ has no interpretable total score.

#### Depressive symptoms

The 4-item Center for Epidemiologic Studies Depression Scale (CESD-4; Melchior et al., 1993) is a shortened version of a revised version of the scale that measures symptoms of depression during the past week. Each depression symptom is followed by a 4-point Likert-type response scale capturing frequency of experiencing that symptom during the past 7 days [0 = rarely or none of the time (<1 day); 1 = some or a little of the time (1 or 2 days); 2 = occasionally or a moderate amount of time (3 or 4 days); 3 = most or all of the time (5–7 days)]. The CESD-4 total score was calculated by summing the four items (theoretical range: 0–12); to obtain unbiased estimates of the total CESD-4 score, idiopathic mean substitution was applied if participants did not answer one of the CESD-4 items.

#### Suicidality and self-harm

Three questions pertaining to suicidality developed for use with a sample of SMA and young adults accessing crisis services (Goldbach et al., 2017) were included the present study. Participants were asked questions about suicidal ideation (“During the past 12 months, did you ever seriously consider attempting suicide?”), suicide attempt (“During the past 12 months, how many times did you actually attempt suicide?”), and self-harm (“During the past 12 months, how many times did you do something to purposely hurt yourself without wanting to die, such as cutting or burning yourself on purpose?”). Suicide attempt and self-harm were transformed into binary variables; participants who had at least one incident of suicide attempt or self-harm in the past 12 months were recoded as 1 (yes), whereas those who had no attempts or self-harm were recoded as 0 (no).

#### Youth problem behaviors

The R-YSR is a 56-item scale used to measure behavioral problems exhibited during the past 6 months (Ivanova et al., 2007). Each behavior is rated on a 3-point scale: 0 = not true, 1 = somewhat or sometimes true, and 2 = very or often true. Following the R-YSR scoring manual, scale scores were created by summing the scores of items qualified as internalizing behaviors (theoretical range: 0–52), externalizing behaviors (theoretical range: 0–44), and total problem behaviors (theoretical range: 0–122).

#### Lifetime and 30-day substance use

Binary items regarding lifetime history of drug and alcohol use were taken from the Youth Risk Behavior Survey (Centers for Disease Control and Prevention, 2010). Participants who endorsed a specific substance were then prompted with a second binary item asking about use of that substance during the past 30 days. Responses were recoded into five binary indicators of lifetime substance use and five additional binary indicators of 30-day substance use, namely alcohol use, tobacco use, marijuana use, prescription drug use (pain relievers, tranquilizers, and stimulants), and use of any other illicit drugs (inhalants, cocaine, synthetic marijuana, poppers, bath salts, heroin, MDMA, GHB, ketamine, LSD, and methamphetamine).

### Analysis

Criterion and divergent validity were initially assessed through analyses of the bivariate relationships between the SMASI total and subscale scores and all validation measures. Specifically, SMASI scores were correlated with CESD-4 total scores; three binary suicidality and self-harm indicators; internalizing, externalizing, and total R-YSR scores; and five lifetime and five 30-day substance use indicators to understand the relationship between minority stress and behavioral and mental health outcomes and to demonstrate criterion validity. Correlations were also examined between SMASI subscale scores and ASQ subscale scores to demonstrate divergent validity. IBM SPSS version 23 was used for correlations when both variables were continuous to produce Pearson correlation coefficients. Mplus version 7 (Muthén and Muthén, 2013) was used to calculate tetrachoric (biserial) correlation coefficients between continuous SMASI scores and dichotomous outcomes (i.e., suicidality, substance use). Given the large number of correlations under investigation, all *p*-values were adjusted using the false discovery rate controlling procedure developed by Benjamini and Hochberg (1995).

Subsequently, criterion validity analysis was conducted in which the constructs underlying the SMASI and ASQ measures (minority stress and general adolescent stress, respectively) were modeled as latent variables in Mplus, wherein their corresponding 10 factors were incorporated as continuous manifest variables. Proximal health outcomes (depressive symptoms, suicidality, and self-harm) and distal health outcomes (youth problem behaviors, substance use) were regressed onto the latent stress variables using robust maximum likelihood estimation. We examined *R*^2^ values for all models with and without the SMASI latent variable to understand the added value of modeling minority stress, beyond general stress, to explain SMA health outcomes.

## Results

### Sample descriptives

Most participants were female (56%) and non-Hispanic White (42%) or Latino or Hispanic (24%). In addition, 43% self-identified as gay, 31% as lesbian, and 27% as bisexual or pansexual; among the bi- or pansexual participants, slightly more were female (63%, *n* = 58) than male (37%, *n* = 34). Nearly one third (30%) of participants reported being currently or previously employed. Roughly one fifth of participants (21%) reported suicidal ideation during the past 12 months, 9% reported at least one suicide attempt, and 32% reported self-harm. A majority of participants (57%) reported using alcohol in their lifetime; a substantial proportion also reported using tobacco (41%), marijuana (28%), and illicit drugs (23%). Complete descriptive statistics for the sample are shown in Table 1.

**Table 1:** Descriptive statistics for the analytic sample (*N* = 346).

**Variable**	***M* (*SD*) or *n* (%)**
**GENDER**	
Male	151 (43.6)
Female	195 (56.4)
**AGE**	
14	35 (10.1)
15	84 (24.3)
16	114 (32.9)
17	113 (32.7)
**RACE**	
Non-Hispanic White	144 (41.6)
Black/African American	40 (11.6)
Latino/Hispanic	84 (24.3)
Asian	28 (8.1)
Other	24 (6.9)
Multiracial	26 (7.5)
**SEXUAL ORIENTATION**	
Gay	147 (42.5)
Lesbian	107 (30.9)
Bisexual/pansexual	92 (26.6)
**EMPLOYMENT STATUS**	
Currently employed	56 (16.2)
Not employed but previously worked	45 (13.0)
Not employed and never worked	244 (70.5)
Suicidal ideation, past 12 months	73 (21.1)
Suicide attempt, past 12 months	32 (9.2)
Self-harm, past 12 months	112 (32.4)
**SUBSTANCE USE (LIFETIME)**	
Alcohol	197 (56.9)
Tobacco	140 (40.5)
Marijuana	98 (28.3)
Prescription drugs	55 (15.9)
Illicit drugs	80 (23.1)
**SUBSTANCE USE (PAST 30 DAYS)**	
Alcohol	112 (35.3)
Tobacco	78 (22.5)
Marijuana	45 (13.0)
Prescription drugs	24 (6.9)
Illicit drugs	39 (11.3)
Depressive symptoms (CESD-4)	5.40 (3.60)
Internalizing behaviors	22.92 (11.85)
Externalizing behaviors	13.97 (8.54)
Total problem behaviors	46.83 (23.45)

### Criterion validity analyses

#### Depressive symptoms

The correlation between the CESD-4 and SMASI total scores was statistically significant for both lifetime (*r* = 0.32, *p* < 0.001) and 30-day (*r* = 0.26, *p* < 0.001) scores (see Table 2). The CESD-4 sum score also demonstrated small to moderate statistically significant correlations with the lifetime SMASI subscales (*r* = 0.17–0.39) and small correlations with the−30-day SMASI subscales (*r* = 0.08–0.24). All but one correlation (with 30-day negative disclosure experiences) were statistically significant.

**Table 2:** Correlations of lifetime and 30-day SMASI with mental health outcomes.

	**Depressive symptoms**	**Suicidal ideation**	**Suicide attempt**	**Self-Harm**
**LIFETIME**				
Whole Scale	0.32^**^	−0.03	0.12	0.14
Social Marginalization	0.21^**^	−0.29^*^	−0.04	0.02
Family Rejection	0.37^**^	0.06	0.10	0.25^*^
Internalized Homonegativity	0.24^**^	−0.15	−0.04	0.06
Identity Management	0.28^**^	−0.04	−0.02	0.02
Homonegative Climate	0.32^**^	−0.06	0.02	0.12
Intersectionality	0.26^**^	−0.12	0.13	0.10
Negative Disclosure Experiences	0.23^**^	−0.21^*^	0.08	0.06
Religion	0.26^**^	−0.03	0.14	0.15
Negative Expectancies	0.17^*^	−0.02	−0.07	0.02
Homonegative Communication	0.39^**^	0.28^*^	0.24	0.29^**^
Work^a^	0.22^*^	−0.05	0.07	0.36^*^
**30-DAY**				
Whole Scale	0.26^**^	0.34^**^	0.29^**^	0.24^**^
Social Marginalization	0.13^*^	−0.02	0.11	0.07
Family Rejection	0.22^**^	0.25^**^	0.19^*^	0.21^*^
Internalized Homonegativity	0.17^*^	0.07	0.10	0.15^*^
Identity Management	0.19^**^	0.16^*^	0.17^*^	0.19^*^
Homonegative Climate	0.21^**^	0.21^*^	0.21^*^	0.18^*^
Intersectionality	0.15^*^	0.16^*^	0.23^*^	0.07
Negative Disclosure Experiences	0.08	−0.07	−0.02	0.09
Religion	0.18^*^	0.22^*^	0.19^*^	0.13
Negative Expectancies	0.12^*^	0.20^*^	0.06	0.08
Homonegative Communication	0.22^**^	0.51^**^	0.38^**^	0.26^**^
Work^a^	0.24^*^	−0.03	0.14	0.40^*^

a *Correlations based on N = 101 participants who reported a history of employment*.

* p < 0.05;

** *p < 0.01*.

#### Suicidality and self-harm

Although the lifetime SMASI total score was not significantly associated with 12-month suicidality or self-harm items, the 30-day SMASI total score was significantly correlated with a history of suicidal ideation (*r* = 0.34, *p* < 0.001), suicide attempt (*r* = 0.29, *p* < 0.001), and self-harm (*r* = 0.24, *p* < 0.001; see Table 2). Similarly, lifetime SMASI factor scores showed small correlations with suicidal ideation (*r* = −0.29 to 0.28), suicide attempt (*r* = −0.07 to 0.24), and self-harm (*r* = 0.02–0.36); however, most of the 30-day SMASI factor scores were significantly correlated with suicidal ideation (*r* = −0.07 to 0.51), suicide attempt (*r* = −0.02 to 0.38), and self-harm (*r* = 0.07–0.40).

#### Youth problem behaviors

Correlations were moderately strong between the lifetime SMASI total score and R-YSR internalizing (*r* = 0.43, *p* < 0.001), externalizing (*r* = 0.60, *p* < 0.001), and total (*r* = 0.53, *p* < 0.001) problem behaviors (see Table 3). Correlations between the 30-day SMASI total score and internalizing (*r* = 0.40, *p* < 0.001), externalizing (*r* = 0.29, *p* < 0.001), and total (*r* = 0.39, *p* < 0.001) problem behavior scores were somewhat reduced, though still statistically significant. Similarly, lifetime SMASI subscale scores had statistically moderate correlations with the R-YSR internalizing (*r* = 0.28–0.52), externalizing (*r* = 0.32–0.57), and total (*r* = 0.34–0.57) problem behavior scores; the 30-day SMASI factors produced smaller correlations with internalizing (*r* = 0.16–0.36), externalizing (*r* = 0.08–0.35), and total (*r* = 0.14–0.35) problem behavior scores than the lifetime SMASI factors.

**Table 3:** Correlations of lifetime and 30-day SMASI with internalizing, externalizing, and total problem behavior scores.

**Sexual minority adolescent stress inventory**	**Internalizing behaviors**	**Externalizing behaviors**	**Total problem behaviors**
**LIFETIME**			
Whole Scale	0.43^**^	0.60^**^	0.53^**^
Social Marginalization	0.28^**^	0.55^**^	0.41^**^
Family Rejection	0.47^**^	0.57^**^	0.55^**^
Internalized Homonegativity	0.33^**^	0.50^**^	0.42^**^
Identity Management	0.33^**^	0.44^**^	0.41^**^
Homonegative Climate	0.36^**^	0.53^**^	0.46^**^
Intersectionality	0.31^**^	0.49^**^	0.41^**^
Negative Disclosure Experiences	0.32^**^	0.47^**^	0.40^**^
Religion	0.33^**^	0.46^**^	0.40^**^
Negative Expectancies	0.29^**^	0.38^**^	0.35^**^
Homonegative Communication	0.52^**^	0.53^**^	0.57^**^
Work^a^	0.33^*^	0.32^*^	0.34^*^
**30-DAY**			
Whole Scale	0.39^**^	0.29^**^	0.39^**^
Social Marginalization	0.27^**^	0.35^**^	0.32^**^
Family Rejection	0.35^**^	0.21^**^	0.33^**^
Internalized Homonegativity	0.26^**^	0.23^**^	0.26^**^
Identity Management	0.23^**^	0.22^**^	0.25^**^
Homonegative Climate	0.30^**^	0.27^**^	0.31^**^
Intersectionality	0.16^*^	0.08	0.14^*^
Negative Disclosure Experiences	0.16^*^	0.18^*^	0.17^*^
Religion	0.26^**^	0.17^*^	0.24^**^
Negative Expectancies	0.21^**^	0.12^*^	0.19^**^
Homonegative Communication	0.31^**^	0.08	0.25^**^
Work^a^	0.36^**^	0.31^*^	0.35^**^

a Correlations based on N = 101 participants who reported a history of employment.

* p < 0.05;

** *p < 0.01*.

#### Lifetime substance use

The lifetime total SMASI score was significantly correlated with lifetime use of alcohol (*r* = 0.16, *p* < 0.05), tobacco (*r* = 0.37, *p* < 0.001), prescription drugs (*r* = 0.33, *p* < 0.001), and illicit drugs (*r* = 0.47, *p* < 0.001), but not marijuana (*r* = 0.01, *p* > 0.05; see Table 4). Five of the 11 lifetime SMASI subscales were correlated with lifetime alcohol use (*r* = 0.16–0.25), namely social marginalization, family rejection, identity management, homonegative climate, and homonegative communication. The main lifetime SMASI subscales were all significantly correlated with lifetime tobacco use (*r* = 0.15–0.36), although the optional work subscale was not. Lifetime marijuana use was only associated with the homonegative communications lifetime factor (*r* = 0.29, *p* < 0.05). All subscales except negative disclosure experiences were significantly correlate with lifetime prescription drug use (*r* = 0.20–0.50). Finally, each of the lifetime SAMSI subscale scores was significantly associated with lifetime illicit drug use (*r* = 0.19–0.44).

**Table 4:** Correlations of lifetime SMASI with lifetime substance use.

**Sexual minority adolescent stress inventory**	**Lifetime alcohol**	**Lifetime tobacco**	**Lifetime marijuana**	**Lifetime prescription**	**Lifetime illicit drugs**
Whole Scale	0.16^*^	0.37^**^	0.01	0.33^**^	0.47^**^
Social Marginalization	0.17^*^	0.35^**^	−0.12	0.20^*^	0.47^**^
Family Rejection	0.17^*^	0.34^**^	0.07	0.35^**^	0.41^**^
Internalized Homonegativity	0.07	0.29^**^	−0.04	0.27^*^	0.44^**^
Identity Management	0.16^*^	0.28^**^	0.05	0.23^*^	0.34^**^
Homonegative Climate	0.19^*^	0.36^**^	0.06	0.38^**^	0.40^**^
Intersectionality	0.08	0.27^**^	−0.01	0.27^*^	0.36^**^
Negative Disclosure Experiences	0.14	0.32^**^	−0.05	0.18	0.38^**^
Religion	0.12	0.33^**^	0.01	0.25^*^	0.41^**^
Negative Expectancies	−0.03	0.15^*^	−0.11	0.27^*^	0.19^*^
Homonegative Communication	0.25^**^	0.36^**^	0.29^*^	0.50^**^	0.36^**^
Work^a^	−0.19	0.01	−0.03	0.30^*^	0.36^*^

a Correlations based on N = 101 participants who reported a history of employment.

* p < 0.05;

** *p < 0.01*.

#### 30-day substance use

The lifetime total SMASI score was also significantly correlated with 30-day use of alcohol (*r* = 0.18, *p* < 0.05), tobacco (*r* = 0.36, *p* < 0.001), prescription drugs (*r* = 0.29, *p* = 0.01), and illicit drugs (*r* = 0.51, *p* < 0.001), but again, not with marijuana (*r* = −0.02, *p* > 0.05; see Table 5). Six of the 11 lifetime SMASI subscales were associated with recent alcohol use (*r* = 0.15–0.21), namely social marginalization, family rejection, identity management, homonegative climate, negative disclosure experiences, and homonegative communication. Again, all lifetime SMASI subscales except work were significantly correlated with recent tobacco use (*r* = 0.21–0.36). Recent use of marijuana was not associated with any lifetime SMASI subscales (*r* = −0.15 to 0.18, *p* >0.05). However, significant associations emerged between recent prescription drug use and lifetime internalized homonegativity (*r* = 0.32, *p* < 0.05), homonegative climate (*r* = 0.39, *p* < 0.01), and homonegative communication (*r* = 31, *p* < 0.05) subscales. As with lifetime illicit drug use, recent illicit drug use was significantly correlated with all lifetime SMASI subscales (*r* = 0.19–0.47).

**Table 5:** Correlations of lifetime and past 30-day SMASI with past 30-day substance use.

**Sexual minority adolescent stress inventory**	**30-Day alcohol**	**30-Day tobacco**	**30-Day marijuana**	**30-Day prescription**	**30-Day illicit drugs**
**LIFETIME**					
Whole Scale	0.18^*^	0.36^**^	−0.02	0.29^*^	0.51^**^
Social Marginalization	0.21^*^	0.34^**^	−0.15	0.21	0.47^**^
Family Rejection	0.15^*^	0.33^**^	0.03	0.26	0.41^**^
Internalized Homonegativity	0.13	0.31^**^	−0.01	0.32^*^	0.44^**^
Identity Management	0.16^*^	0.29^**^	0.04	0.18	0.34^**^
Homonegative Climate	0.20^*^	0.36^**^	0.02	0.39^*^	0.40^**^
Intersectionality	0.14	0.23^*^	−0.01	0.18	0.36^**^
Negative Disclosure Experiences	0.16^*^	0.33^**^	−0.08	0.17	0.38^**^
Religion	0.11	0.21^*^	0.05	0.22	0.41^**^
Negative Expectancies	0.08	0.26^**^	−0.05	0.09	0.19^*^
Homonegative Communication	0.16^*^	0.36^**^	0.18	0.34^*^	0.36^**^
Work^a^	−0.21	0.00	0.02	0.31	0.36^*^
***30-Day***					
Whole Scale	0.06	0.15	−0.03	0.10	0.08
Social Marginalization	0.11	0.20^**^	−0.14	0.12	0.11
Family Rejection	0.05	0.07	−0.06	0.00	0.05
Internalized Homonegativity	0.01	0.15	0.00	0.15	0.01
Identity Management	0.03	0.18^*^	0.04	0.04	0.03
Homonegative Climate	−0.01	0.10	−0.10	0.12	−0.01
Intersectionality	0.01	0.06	−0.02	0.02	0.01
Negative Disclosure Experiences	0.07	0.11	−0.19	0.08	0.07
Religion	0.02	0.09	0.10	−0.03	0.02
Negative Expectancies	0.04	0.01	−0.13	0.03	0.04
Homonegative Communication	0.03	0.03	0.22^*^	0.10	0.03
Work^a^	−0.34^*^	−0.20	−0.11	0.30^*^	−0.34

a Correlations based on N = 101 participants who reported a history of employment.

* p < 0.05;

** *p < 0.01*.

In contrast to the lifetime SMASI results, the 30-day total SMASI score was not significantly correlated with any 30-day use of alcohol, tobacco, marijuana, prescription drugs, or illicit drugs. Recent alcohol and prescription drug use were associated with only the optional 30-day work subscale of the SMASI (*r* = −0.34, *p* < 0.05; *r* = 0.30, *p* < 0.05, respectively). Recent tobacco use was only significantly correlated with 30-day social marginalization (*r* = 0.20, *p* < 0.001) and identity management (*r* = 0.18, *p* < 0.05) subscales. Recent use of marijuana was significantly correlated with only 30-day homonegative communication subscale (*r* = 0.22, *p* < 0.05). In contrast to the lifetime results, recent illicit drug use was not significantly correlated with any 30-day SMASI subscales.

### Divergent validity analyses

#### General stress

Comparisons between the ASQ subscales and the lifetime (*r* = −0.27 to 0.51) and 30-day (*r* = −0.06 to 0.44) SMASI subscales revealed small to moderate correlations across all pairs of subscales (see Tables 6, 7). All ASQ subscales except for the stress of future uncertainty subscale were significantly correlated with the lifetime total SMASI score (*r* = 0.12–0.50), and 86 of the 110 possible correlations between ASQ subscales and lifetime SMASI subscales were statistically significant. Similarly, ASQ subscales were significantly correlated with the 30-day total SMASI score (*r* = 0.17–0.43), and again 86 of 110 correlations between ASQ subscales and past 30-day SMASI subscales were significant. These small to moderate correlations between sexual minority stress and general adolescent stress show good divergent validity; whereas the two underlying constructs both measure stress, the low magnitude of the correlations across pairs of subscales demonstrates relatively minimal overlap in measurement between the SMASI and this validated measure of general adolescent stress.

**Table 6:** Correlations between lifetime SMASI and 12-month general stress factors.

**Sexual minority adolescent stress inventory**	**Home life**	**School perf**.	**School attend**.	**Romant. rships**	**Peer press**.	**Teacher interact**.	**Future unc**.	**S/L conflict**	**Financ. press**.	**Adult resp**.
Whole Scale	0.26^**^	0.15^*^	0.12^*^	0.30^**^	0.39^**^	0.50^**^	−0.09	0.19^*^	0.23^**^	0.37^**^
Social Marginalization	0.05	0.03	−0.01	0.19^*^	0.29^**^	0.43^**^	−0.27^**^	0.04	0.13^*^	0.30^**^
Family Rejection	0.39^**^	0.17^*^	0.15^*^	0.32^**^	0.37^**^	0.42^**^	−0.02	0.22^**^	0.28^**^	0.36^**^
Internalized Homonegativity	0.11	0.05	0.07	0.20^**^	0.27^**^	0.34^**^	−0.13^*^	0.10	0.11	0.28^**^
Identity Management	0.16^*^	0.14^*^	0.14^*^	0.27^**^	0.31^**^	0.34^**^	0.01	0.11	0.13^*^	0.20^**^
Homonegative Climate	0.22^**^	0.16^*^	0.13^*^	0.27^**^	0.38^**^	0.51^**^	−0.13^*^	0.14^*^	0.24^**^	0.34^**^
Intersectionality	0.27^**^	0.14^*^	0.10	0.22^**^	0.32^**^	0.45^**^	−0.02	0.27^**^	0.21^**^	0.40^**^
Negative Disclosure Experiences	0.23^**^	0.14^*^	0.08	0.25^**^	0.35^**^	0.42^**^	−0.07	0.17^*^	0.20^**^	0.31^**^
Religion	0.39^**^	0.13^*^	0.10	0.19^*^	0.29^**^	0.37^**^	−0.04	0.21^**^	0.21^**^	0.27^**^
Negative Expectancies	0.18^*^	0.17^*^	0.17^*^	0.25^**^	0.38^**^	0.41^**^	−0.05	0.18^*^	0.13^*^	0.31^**^
Homonegative Communication	0.44^**^	0.28^**^	0.24^**^	0.35^**^	0.41^**^	0.43^**^	0.28^**^	0.36^**^	0.33^**^	0.33^**^
Work^a^	0.15	0.05	0.06	0.10	0.22^*^	0.27^*^	−0.06	0.06	0.18	0.23^*^

a Correlations based on N = 101 participants who reported a history of employment.

* p < 0.05;

** *p < 0.01*.

**Table 7:** Correlations between past 30-day SMASI and 12-month general stress factors.

**Sexual minority adolescent stress inventory**	**Home life**	**School perf**.	**School attend**.	**Romant. rships**	**Peer press**.	**Teacher interact**.	**Future unc**.	**S/L conflict**	**Financ. press**.	**Adult resp**.
Whole Scale	0.43^**^	0.23^**^	0.25^**^	0.23^**^	0.39^**^	0.39^**^	0.17^*^	0.31^**^	0.35^**^	0.39^**^
Social Marginalization	0.14^*^	0.07	0.08	0.14^*^	0.27^**^	0.36^**^	−0.06	0.07	0.21^**^	0.34^**^
Family Rejection	0.45^**^	0.18^*^	0.20^**^	0.28^**^	0.29^**^	0.28^**^	0.17^*^	0.31^**^	0.33^**^	0.33^**^
Internalized Homonegativity	0.17^*^	0.10	0.08	0.10	0.27^**^	0.20^**^	0.00	0.15^*^	0.13^*^	0.22^**^
Identity Management	0.20^*^	0.12^*^	0.20^**^	0.16^*^	0.29^**^	0.22^**^	0.09	0.09	0.13^*^	0.18^*^
Homonegative Climate	0.19^*^	0.14^*^	0.18^*^	0.06	0.28^**^	0.35^**^	−0.02	0.16^*^	0.22^**^	0.28^**^
Intersectionality	0.36^**^	0.17^*^	0.16^*^	0.11	0.23^**^	0.27^**^	0.23^**^	0.32^**^	0.24^**^	0.33^**^
Negative Disclosure Experiences	0.14^*^	0.02	0.05	0.07	0.16^*^	0.25^**^	−0.12^*^	0.03	0.19^*^	0.18^*^
Religion	0.34^**^	0.17^*^	0.22^**^	0.14^*^	0.21^**^	0.25^**^	0.17^*^	0.26^**^	0.23^**^	0.18^*^
Negative Expectancies	0.26^**^	0.27^**^	0.22^**^	0.18^*^	0.32^**^	0.25^**^	0.15^*^	0.26^**^	0.19^**^	0.21^**^
Homonegative Communication	0.45^**^	0.28^**^	0.27^**^	0.17^*^	0.30^**^	0.19^**^	0.44^**^	0.34^**^	0.35^**^	0.25^**^
Work^a^	0.13	0.08	0.04	0.11	0.30^*^	0.36^**^	−0.04	0.14	0.17	0.33^*^

a Correlations based on N = 101 participants who reported a history of employment.

* p < 0.05;

** *p < 0.01*.

### Explanatory utility beyond general stress

#### Lifetime minority stress and general adolescent stress

The measurement model of lifetime minority stress estimated the factor loadings of each of the main 10 factors (subscales) on the minority stress latent construct; to avoid restricting the sample size, the optional work factor was excluded. Correlated residuals were added to the model based on modification indices; correlations that provided a chi-square reduction <10 were added individually to each succeeding model. The final model consisted of six correlated residuals and resulted in good fit (CFI = 0.988, TLI = 0.981, RMSEA = 0.059). Factor loadings were moderate to high for all 10 factors (0.64–0.94). A similar process was repeated for general adolescent stress, with the 10 factors of the ASQ loading onto the latent construct of general stress. The final general stress model included five correlated residuals and also resulted in good fit (CFI = 0.966, TLI = 0.949, RMSEA = 0.074), with moderate to high factor loadings (0.54–81). Table 8 displays the factor loadings for the measurement models of sexual minority stress and general adolescent stress.

**Table 8:** Measurement model factor loadings of the SMASI and ASQ subscales.

	**SMASI**	**ASQ**
S1: Social Marginalization	0.94	
S2: Family Rejection	0.91	
S3: Internalized Homonegativity	0.81	
S4: Identity Management	0.66	
S5: Homonegative Climate	0.86	
S6: Intersectionality	0.81	
S7: Negative Disclosure Experiences	0.83	
S8: Religion	0.79	
S9: Negative Expectancies	0.64	
S10: Homonegative Communication	0.72	
A1: Stress of Home Life		0.80
A2: Stress of School Performance		0.81
A3: Stress of School Attendance		0.74
A4: Stress of Romantic Relationships		0.54
A5: Stress of Peer Pressure		0.72
A6: Stress of Teacher Interaction		0.67
A7: Stress of Future Uncertainty		0.61
A8: Stress of School/Leisure Conflict		0.76
A9: Stress of Financial Pressure		0.72
A10: Stress of Adult Responsibility		0.56

#### General approach to structural models

**Figure 1:**
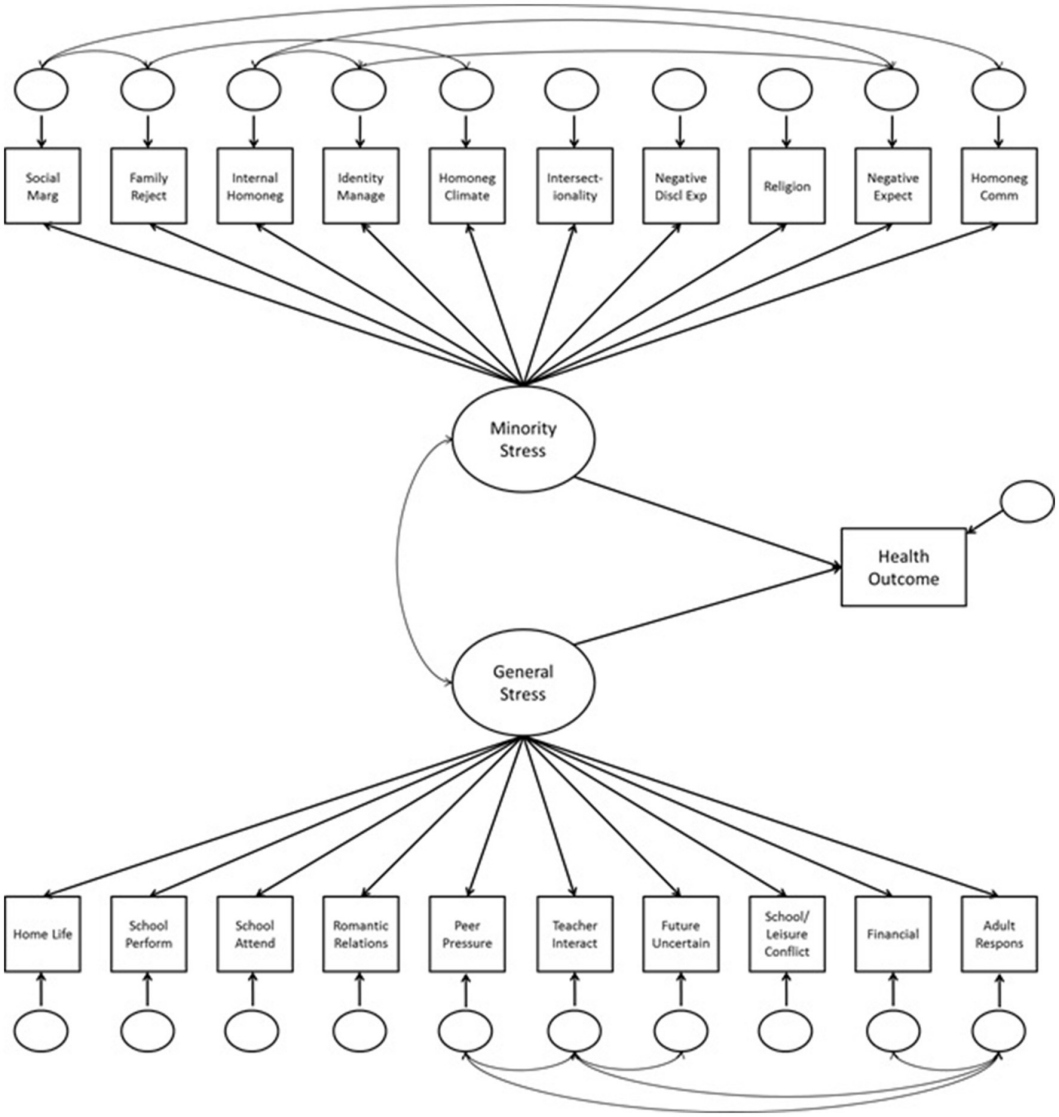
Structural model for divergent validity analyses.

In all structural models, we first assessed the relationship between general stress and each health outcome (depressive symptoms, suicidality and self-harm, youth problem behaviors, and 30-day substance use). The minority stress latent variable was then included to assess the change in predictive utility of the model with and without minority stress. Because the minority and general stress latent constructs were significantly correlated (*r* = 0.27, *p* < 0.001), this correlation was included in the structural models incorporating both constructs (Figure 1).

#### Proximal health outcomes: depressive symptoms, suicidality, self-harm

The minority stress latent construct was significantly associated with depressive symptoms (β = 0.21, *p* < 0.001) and suicidal ideation (β = −0.24, *p* < 0.001). General stress was associated with depressive symptoms (β = 0.49, *p* < 0.001), suicidal ideation (β = 0.61, *p* < 0.001), suicide attempt (β = 0.51, *p* < 0.001), and self-harm (β = 0.37, *p* < 0.001).

#### Distal health outcomes: youth problem behaviors, substance use

Minority stress was significantly associated with internalizing behaviors (β = 0.28, *p* < 0.001), externalizing behaviors (β = 0.57, *p* < 0.001), and total problem behaviors (β = 0.41, *p* < 0.001). Furthermore, minority stress was significantly associated with 30-day use of alcohol (β = 0.19, *p* < 0.01), tobacco (β = 0.37, *p* < 0.001), prescription drugs (β = 0.32, *p* < 0.05), and illicit drugs (β = 0.67, *p* < 0.01), but not marijuana (β = −0.09, *p* > 0.05). General stress was also significantly associated with internalizing (β = 0.57, *p* < 0.001), externalizing (β = 0.18, *p* < 0.001), and total (β = 0.49, *p* < 0.001) problem behaviors; however, general stress was only positively associated with 30-day marijuana use (β = 0.21, *p* < 0.05) and demonstrated a strong negative relationship with 30-day illicit drug use (β = −0.81, *p* < 0.001).

Table 9 presents the *R*^2^ values for all outcomes, from models with general stress alone (ASQ) to models including both general and minority stress (ASQ and SMASI). For all outcomes except marijuana use, suicide attempt, and self-harm, the inclusion of the SMASI-based minority stress latent factor increased the explanatory power of the model, including substantially for some outcomes (e.g., externalizing and total problem behaviors; tobacco, prescription drug, and illicit drug use).

**Table 9:** *R*^*2*^ values for health outcomes based on modeled associations with general stress alone (ASQ) versus both general stress and minority stress (ASQ + SMASI).

	***R*^2^**	
	**ASQ**	**ASQ + SMASI**
Depressive symptoms	0.294	0.335
Suicidal ideation	0.335	0.350
Suicide attempt	0.255	0.255
Self-harm	0.145	0.152
Internalizing behaviors	0.413	0.485
Externalizing behaviors	0.109	0.414
Total problem behaviors	0.357	0.515
30-day alcohol use	0.002	0.036
30-day tobacco use	0.009	0.139
30-day marijuana use	0.036	0.041
30-day prescription drug use	0.003	0.102
30-day illicit drug use	0.190	0.720

## Discussion

It has been nearly 15 years since the seminal minority stress theory was formally described by Meyer (2003). In that span, numerous studies, including meta-analyses, have established evidence of significant health disparities for SMA as compared to their peers (Marshal et al., 2008, 2011; Friedman et al., 2011) and dozens of studies have attributed these outcomes to the presence of minority stress. Indeed, it is difficult to find a study of health disparities that does not cite minority stress theory (3,652 citations as of March 2017). Yet as Morrison et al. (2016) found in their review, the ability to accurately measure minority stress has been hindered by the lack of sound psychometric instrumentation. Our study adds to the literature in three key ways: (a) we validated the first comprehensive measure of minority stress for adolescents; (b) we added evidence of the relationship between minority stress and poor behavioral health patterns; and (c) we found evidence that minority stress is indeed different than general adolescent stress experiences.

To our knowledge, this study provides the field with the first validated measure of minority stress for adolescents, including 10 key subscales of stress. Aligning with minority stress theory, these subscales include distal stressors (i.e., social marginalization, family rejection, homonegative climate, negative disclosure experiences, religious conflict, homonegative communication) and proximal stressors (i.e., internalized homonegativity, identity management, intersectionality, negative expectancies). Valid and reliable measurement is a necessary antecedent in the development of explanatory research and intervention efforts (Sandler et al., 1997) and accurate clinical assessment and appraisal of mental health needs (Watkins et al., 1995; Groth-Marnat, 2009). We expect that the resulting measure therefore may have implications for clinical screening and could provide a foundation for future intervention efforts that target these 10 key domains as potential mechanisms of change.

To the second point, both lifetime and recent (30-day) experiences of minority stress were associated with a myriad of behavioral health outcomes including symptoms of depression, suicidality and self-harm (30-day only), problem behaviors (internalizing and externalizing), and substance use. These associations largely align with previous work. With regard to depression, recent meta-analytic results showed that SMA have higher levels of depressive symptoms than their heterosexual peers (Marshal et al., 2011). Similarly, recent studies found that SMA are 3 to 4 times more likely to meet criteria for an internalizing disorder (Kann, 2011). Our study provides new methods for understanding the unique processes occurring for SMA related to these differences.

In the case of substance use, we found somewhat less consistent relationships with the SMASI. Although lifetime experiences of minority stress were associated with both lifetime and 30-day alcohol, tobacco, prescription drug, and illicit drug use, recent experiences of minority stress as measured by the 30-day SMASI were not. Furthermore, neither lifetime nor 30-day minority stress was associated with marijuana use. With regard to marijuana use, the majority of participants in this sample resided in states where marijuana use has been decriminalized and is increasingly socially acceptable among youth (CHKS, 2015). Thus stress may have no impact on use patterns. Regarding the relationship between recent minority stress and substance use, we contend that perhaps the production of these distal outcomes (drug use) take time to manifest. For example, SMA are more likely to be abused by their family and peers, with particularly high rates of severe violence such as being accosted by a peer with a weapon at school (Friedman et al., 2011). Youth who are rejected by parents or peers may subsequently develop deviant peer relationships that lead to antisocial behaviors such as substance use (Marshal and Molina, 2006). Further exploration of these extraneous factors (e.g., deviant peer relationships) that may explain why lifetime but not recent reporting of minority stress was associated with recent substance use is warranted, particularly with a longitudinal design.

Perhaps the most important way our study adds to the literature base for minority stress is with respect to our divergent validity analyses. A common argument against the minority stress hypothesis is that “stress is bad for everyone” and that the characterization of a stressor as minority specific is unnecessary. By this logic, family rejection would have the same impact on a young person's behavioral health as family rejection for being a sexual minority. Our study suggests that there is indeed a difference between these two types of stress. We established that although the two underlying constructs (general stress, as measured by the ASQ, and minority stress, as measured by the SMASI) both capture stress experiences, the low magnitude of correlations between these measures indicates that the SMASI is not duplicative of a general stress measure. Further, including a measure of minority stress adds explanatory power beyond that of a general stress measure, especially in the case of externalizing behaviors and substance use patterns. Implications for research and practice are evident: By not measuring minority stress experiences among SMA, we may be missing key factors that underlie their behavioral health profile.

### Limitations and conclusion

Certainly, the findings of this study should be considered in light of limitations. Our data were cross-sectional, and therefore associations between stress and behavioral health should not be seen as causal. Given the limited longitudinal research with this population, expanding prospective research is a critical next step. Second, all measures in the present study were self-reported and thus subject to potential response bias. Similarly, while we employed respondent driven sampling (Heckathorn, 1997) to reach participants outside our local area, our sample nonetheless may not fully generalize to the entire population of U.S. sexual minority youth. Therefore, whether the SMASI would perform differently with harder to reach youth (e.g., those not out to anyone) is difficult to determine. Future studies should include biological and observed markers of stress to refine our understanding of the minority stress-illness relationship. Finally, the validation of the final 64-item measure was derived from an initial set of 102 candidate items described elsewhere (blinded for review). Thus, it is possible that responses to these items may change when only the final set of items is asked of participants.

Our objective for the present study was to validate a measure of minority stress for SMA. The final SMASI—in its overall form and each of its 11 subscales—was associated with nearly all of the outcomes, as minority stress theory would suggest. Yet, while our study met its aims, it also uncovers new questions that could not be explored. For example, even among SMA, there are disparities by demographic subgroup; meta-analyses find sexual minority girls are more likely to report both considering and attempting suicide than sexual minority boys (Friedman et al., 2011), and bisexual youth report larger substance use disparities than other groups (Marshal et al., 2009). Given constraints on statistical power for subgroup analysis when developing the SMASI, we were not able to fully examine differences by all racial or ethnic minority subgroups, despite the diverse sample we were able to recruit. There are known differences in outcomes among SMA by race, ethnicity (Moradi et al., 2009), and urbanicity or international setting (Cohn and Leake, 2012) that could not be controlled for in the present analysis and may potentially represent confounding by stress attributable to another minority status, such as race or ethnicity. Whether differences in reporting of minority stress may account for these differences in outcomes remains an open question and larger samples that can lead to multi-group analyses across gender and sexual identity subgroups, race, ethnicity and urbanicity are a necessary next step.

Given the significant disparities found in this population of young people, we hope that the validated measure can be used to enhance future research and the development of practical applications for this high-need population, including a comprehensive test of the minority stress model cross-sectionally and longitudinally. Cross-sectional analyses within a structural equation modeling framework are already underway based on the current study's dataset, and longitudinal data using the new SMASI measure are forthcoming. Because sound psychometric measurement is a cornerstone of intervention development, we suspect that these will be only the first steps in developing a more precise understanding of the stress and illness relationship found among SMA, and that future studies will build on this foundation of measurement science.

## Ethics statement

This study was carried out in accordance with the human subjects protection guidelines of the National Institutes of Health (NIH) and the principles of the Declaration of Helsinki. All participants in this observational survey study were minors between the ages of 14–17, and therefore provided assent to participate by verbally assenting to study procedures after reading a paper assent form document (if recruited in person) or by indicating their assent with a binary survey item after reading the online assent form outlining study procedures (if recruited online). Given that participants were sexual minorities (e.g., lesbian, gay, or bisexual) who may not have previously disclosed their orientation to their parents, and thus notifying parents about their study participation could have placed participants at additional risk, a waiver of parental consent was granted by the approving IRBs. We also obtained a Certificate of Confidentiality from the NIH. The study protocol was approved by the Institutional Review Boards of Children's Hospital Los Angeles and the University Park campus of the University of Southern California.

## Author contributions

JG and SS jointly conceptualized the study reported in this manuscript. JG was responsible for participant recruitment and data collection, drafted the initial manuscript, and critically revised the manuscript. SS was responsible for data management, developed the analytic plan, and critically revised the manuscript. MM assisted with data analysis, contributed to the statistical analysis and results sections, and critically reviewed the manuscript. All authors approved the final manuscript as submitted.

### Conflict of interest statement

The authors declare that the research was conducted in the absence of any commercial or financial relationships that could be construed as a potential conflict of interest.
