# Supplementary material for: Criterion and Divergent Validity of the Sexual Minority Adolescent Stress Inventory
Source: Front Psychol. 2017 Nov 28;8:2057. doi: 10.3389/fpsyg.2017.02057 (PMC5712417; doi:10.3389/fpsyg.2017.02057)
Supplement: Supplementary file 1 [file Table1.pdf]

The Sexual Minority Adolescent Stress Inventory (SMASI)

We'd like to understand more about stress experienced by LGBTQ youth. This survey includes statements that reflect thoughts, feelings and experiences that may be happening to you now or have happened sometime in the past. Some questions and statements have different instructions so please read each of these instructions carefully. There are no right or wrong answers.

Below are statements that reflect different types of stressful thoughts or events that you may have experienced. Please read each statement and answer "Yes" if it has ever happened to you in the past, or "No" if it hasn't. If you said "Yes" to a statement, please also answer the follow-up question about whether it is currently happening. For the follow-up questions, you should answer "Yes" if it happened to you within the past 30 days, or "No" if it happened to you more than 30 days ago.

You should select the one option that best represents your experience for each statement.

	Yes	No
1. I am questioning how to label my sexual orientation.	◯	◯
└→ **IF YES**: was it within the past 30 days?	◯	◯
2. I am having trouble accepting that I am LGBTQ.	◯	◯
└→ **IF YES**: was it within the past 30 days?	◯	◯
3. I feel pressured to label myself as gay or lesbian.	◯	◯
└→ **IF YES**: was it within the past 30 days?	◯	◯
4. I am concerned that if I am LGBTQ, I will have a worse life than if I were straight.	◯	◯
└→ **IF YES**: was it within the past 30 days?	◯	◯
5. A family member told other family members that I am LGBTQ without my permission.	◯	◯
└→ **IF YES**: was it within the past 30 days?	◯	◯
6. A family member told me not to tell other family members that I am LGBTQ.	◯	◯
└→ **IF YES**: was it within the past 30 days?	◯	◯
7. I have to lie to my family about being LGBTQ.	◯	◯
└→ **IF YES**: was it within the past 30 days?	◯	◯
8. I think I will lose friends if I come out as LGBTQ.	◯	◯
└→ **IF YES**: was it within the past 30 days?	◯	◯
9. I expect people to reject me when they find out that I am LGBTQ.	◯	◯
└→ **IF YES**: was it within the past 30 days?	◯	◯
10. If I come out, it will cause problems within my family.	◯	◯
└→ **IF YES**: was it within the past 30 days?	◯	◯
11. A family member asked me if I was gay or lesbian before I wanted to talk about it.	◯	◯
└→ **IF YES**: was it within the past 30 days?	◯	◯
12. I was forced to come out to someone because I got "caught".	◯	◯
└→ **IF YES**: was it within the past 30 days?	◯	◯
13. I was "outed" by someone other than my family without my permission.	◯	◯
└→ **IF YES**: was it within the past 30 days?	◯	◯
14. There are times when I do not want to be LGBTQ.	◯	◯
└→ **IF YES**: was it within the past 30 days?	◯	◯
15. If I could, I would become straight.	◯	◯
└→ **IF YES**: was it within the past 30 days?	◯	◯

	Yes	No
16. I hate being LGBTQ.	◯	◯
└→ **IF YES**: was it within the past 30 days?	◯	◯
17. I think it is wrong for me to be LGBTQ.	◯	◯
└→ **IF YES**: was it within the past 30 days?	◯	◯
18. I hope that being LGBTQ is just a phase for me.	◯	◯
└→ **IF YES**: was it within the past 30 days?	◯	◯
19. I think negatively about other LGBTQ people who act "too gay".	◯	◯
└→ **IF YES**: was it within the past 30 days?	◯	◯
20. I am uncomfortable with being LGBTQ.	◯	◯
└→ **IF YES**: was it within the past 30 days?	◯	◯
21. I have heard a family member make negative comments about LGBTQ people.	◯	◯
└→ **IF YES**: was it within the past 30 days?	◯	◯
22. My family does not want to talk to me about being LGBTQ.	◯	◯
└→ **IF YES**: was it within the past 30 days?	◯	◯
23. Someone who lives with me has told me they disapprove of me being LGBTQ.	◯	◯
└→ **IF YES**: was it within the past 30 days?	◯	◯
24. I feel as though I am a disappointment to my family because I am LGBTQ.	◯	◯
└→ **IF YES**: was it within the past 30 days?	◯	◯
25. My family has told me that being LGBTQ is just a phase.	◯	◯
└→ **IF YES**: was it within the past 30 days?	◯	◯
26. My parents are uncomfortable with LGBTQ people.	◯	◯
└→ **IF YES**: was it within the past 30 days?	◯	◯
27. My mother (or female caregiver) does not accept me as LGBTQ.	◯	◯
└→ **IF YES**: was it within the past 30 days?	◯	◯
28. My father (or male caregiver) does not accept me as LGBTQ.	◯	◯
└→ **IF YES**: was it within the past 30 days?	◯	◯
29. My parents are sad that I am LGBTQ.	◯	◯
└→ **IF YES**: was it within the past 30 days?	◯	◯
30. My family tries to make me straight.	◯	◯
└→ **IF YES**: was it within the past 30 days?	◯	◯
31. I felt unsafe or threatened in school because I am LGBTQ.	◯	◯
└→ **IF YES**: was it within the past 30 days?	◯	◯
32. Other youth refuse to do school activities with me because I am LGBTQ.	◯	◯
└→ **IF YES**: was it within the past 30 days?	◯	◯
33. I have seen other LGBTQ youth treated badly at my school.	◯	◯
└→ **IF YES**: was it within the past 30 days?	◯	◯
34. It's hard to be an LGBTQ person at my school.	◯	◯
└→ **IF YES**: was it within the past 30 days?	◯	◯
35. Other students make fun of me for being LGBTQ.	◯	◯
└→ **IF YES**: was it within the past 30 days?	◯	◯
36. I have seen other LGBTQ youth treated badly in the neighborhood where I live.	◯	◯
└→ **IF YES**: was it within the past 30 days?	◯	◯

	Yes	No
37. I have felt unsafe or threatened in the neighborhood where I live because I am LGBTQ.	◯	◯
└→ **IF YES**: was it within the past 30 days?	◯	◯
38. I have had to move or change where I live because I am LGBTQ.	◯	◯
└→ **IF YES**: was it within the past 30 days?	◯	◯
39. I have felt isolated or alone in the neighborhood where I live because I am LGBTQ.	◯	◯
└→ **IF YES**: was it within the past 30 days?	◯	◯
40. Other people in the neighborhood where I live make fun of me for being LGBTQ.	◯	◯
└→ **IF YES**: was it within the past 30 days?	◯	◯
41. I have been physically assaulted in the neighborhood where I live because I am LGBTQ.	◯	◯
└→ **IF YES**: was it within the past 30 days?	◯	◯
42. My friends make jokes about LGBTQ people.	◯	◯
└→ **IF YES**: was it within the past 30 days?	◯	◯
43. Other youth refuse to hang out with me because I am LGBTQ.	◯	◯
└→ **IF YES**: was it within the past 30 days?	◯	◯
44. Other people who are in my racial/ethnic community judge me for being LGBTQ.	◯	◯
└→ **IF YES**: was it within the past 30 days?	◯	◯
45. I have heard negative comments from others in my racial/ethnic community about being LGBTQ.	◯	◯
└→ **IF YES**: was it within the past 30 days?	◯	◯
46. I feel as though I don't fit in my racial/ethnic community because I am LGBTQ.	◯	◯
└→ **IF YES**: was it within the past 30 days?	◯	◯
47. As an LGBTQ person in my racial/ethnic community, I feel like I am a minority within a minority.	◯	◯
└→ **IF YES**: was it within the past 30 days?	◯	◯
48. I hear other LGBTQ people use words like "fag" or "dyke."	◯	◯
└→ **IF YES**: was it within the past 30 days?	◯	◯
49. My family is part of a religion that has homophobic beliefs.	◯	◯
└→ **IF YES**: was it within the past 30 days?	◯	◯
50. I have heard negative messages about being LGBTQ from religious people.	◯	◯
└→ **IF YES**: was it within the past 30 days?	◯	◯
51. I would not be accepted as an LGBTQ person in my family's religion.	◯	◯
└→ **IF YES**: was it within the past 30 days?	◯	◯
52. I believe it is wrong for me to be LGBTQ because of my religion.	◯	◯
└→ **IF YES**: was it within the past 30 days?	◯	◯
53. A religious leader has encouraged me to reconsider my sexual orientation.	◯	◯
└→ **IF YES**: was it within the past 30 days?	◯	◯
54. A religious leader tried to change my sexual orientation.	◯	◯
└→ **IF YES**: was it within the past 30 days?	◯	◯

Please also answer the following if you are currently, or have previously been, employed.

	Yes	No
55. I have seen other LGBTQ youth treated badly at work.	◯	◯
└→ **IF YES**: was it within the past 30 days?	◯	◯
56. I have felt unsafe or threatened at work because I am LGBTQ.	◯	◯
└→ **IF YES**: was it within the past 30 days?	◯	◯
57. I have had to leave or change jobs because I am LGBTQ.	◯	◯
└→ **IF YES**: was it within the past 30 days?	◯	◯
58. I have felt isolated or alone at work because I am LGBTQ.	◯	◯
└→ **IF YES**: was it within the past 30 days?	◯	◯
59. I have lost friendships since coming out as LGBTQ at work.	◯	◯
└→ **IF YES**: was it within the past 30 days?	◯	◯
60. It's hard to be LGBTQ at my workplace.	◯	◯
└→ **IF YES**: was it within the past 30 days?	◯	◯
61. I have been physically assaulted by people at work because I am LGBTQ.	◯	◯
└→ **IF YES**: was it within the past 30 days?	◯	◯
62. My workplace does not protect LGBTQ employees.	◯	◯
└→ **IF YES**: was it within the past 30 days?	◯	◯
63. People at work talk about me being LGBTQ behind my back.	◯	◯
└→ **IF YES**: was it within the past 30 days?	◯	◯
64. My boss is unsupportive of me because I am LGBTQ.	◯	◯
└→ **IF YES**: was it within the past 30 days?	◯	◯

Scoring Instructions for the SMASI

Four types of scores can be created based on a complete SMASI instrument: overall lifetime score (items 1 – 54 only), overall 30-day score (items 1 – 54 only), subscale lifetime scores, and subscale 30-day scores.

Overall

Responses to lifetime (i.e., numbered) items are scored in a binary fashion: “Yes” responses are coded as 1, “No” responses are coded as 0. The coded responses to items 1 – 54 are summed to create the overall lifetime score (theoretical range: 0 to 54). A similar procedure is used for the supplemental 30-day (i.e., “IF YES”) items to create the overall 30-day score (theoretical range: 0 to 54). Idiographic mean substitution is recommended for participants who skipped or declined to answer individual items

Subscales

Lifetime and 30-day subscale scores are created as percentages of endorsed statements within the given subscale. Correspondence between subscales and item numbers is as follows:

Identity management: Items 1, 2, 3

Negative expectancies: Items 4, 8, 9

Negative disclosure experiences: Items 5, 6, 11, 12, 13

Family rejection: Items 7, 10, 22, 23, 24, 25, 26, 27, 28, 29, 30

Internalized homonegativity: Items 14, 15, 16, 17, 18, 19, 20

Homonegative communication: Items 21, 42, 45, 48, 50

Homonegative climate: Items 31, 33, 34, 35

Social marginalization: Items 32, 36, 37, 38, 39, 40, 41, 43

Intersectionality: Items 44, 46, 47

Religion: Items 49, 51, 52, 53, 54

Work: Items 55, 56, 57, 58, 59, 60, 61, 62, 63, 64

Note: Scores on the work subscale should only be calculated for participants who indicated current or previous employment. Participants who have never been employed should not be given a score on the work subscale, even if they respond to the items.
